# The impact of green credit on economic growth—The mediating effect of environment on labor supply

**DOI:** 10.1371/journal.pone.0257612

**Published:** 2021-09-21

**Authors:** Cai Chen, Yingli Zhang, Yun Bai, Wenrui Li

**Affiliations:** School of Economics and Management, Shanghai Ocean University, Shanghai, China; China University of Mining and Technology, CHINA

## Abstract

**Background:**

The progress of green credit in China is accelerating, but its development is uneven and insufficient in different regions. And whether the issuance of green credit can effectively promote the improvement of the environment and economy is not well understood.

**Objective:**

Previous research has found that green credit promotes economic growth through improvement of the industrial structure and green technological innovation. However, these studies have not considered the positive externality of environmental improvement even though environmental improvement and economic growth are requirements of the sustainable development concept.

**Methods:**

We use the chain-mediated model to estimate the impact of green credit issuance on the economic growth of different provinces since the large-scale implementation of green credit in China with data from 2008 to 2016.

**Results and conclusion:**

This paper shows that the issuance of green credit can improve labor supply rather than labor productivity through the improvement of air quality to achieve regional economic growth. Such a chain-mediated path is different from the economic growth caused by industrial structural adjustment and green technology innovation. At the national level, every 1% increase in green credit issuance relative to industrial loans will increase the per capita gross domestic product (GDP) by approximately 4.6 yuan, or 0.012%, through air quality and labor supply, accounting for 2.875% of the total effect. Heterogeneity analysis indicates that due to regional industrial structure differences and diminishing marginal effects, the impact of green credit is stronger in the western region than in the eastern and central regions. For every 1% increase in the proportion of green credit issuance relative to industrial loans, the per capita GDP growth achieved through the chain-mediated path is approximately 30.17 yuan in the western region, approximately 6.6 times greater than that at the national level. Within a 95% confidence interval of 5000 bootstrap samples, this path is found to be true, and the chain-mediated effect accounts for approximately 12.96% of the total indirect effect.

**Limitations:**

The limitation of this paper is the measurement of green credit. Although green credit has a large volume, it remains underdeveloped, and there is a lack of perfect indicators. Most existing studies have adopted only alternative or reverse indicators to measure the issuance of green credit. For example, this paper takes the interest expenditure of six high-energy-consuming enterprises as the reverse indicator, which may to a certain extent lead to the overestimation of the issuance of green credit and its impact on the environment and economy. Future research can accurately explore the performance of green credit on the basis of its mature development.

## Introduction

Since the reform and opening up, China’s economic development has achieved remarkable results, but economic development has also caused environmental problems. For example, the smog caused by worsening air quality covers an area of more than 1.3 million square kilometers [1], accounting for 13.54% of the land area in China. The main source of smog pollution is PM_2.5_, and the deterioration of air quality caused by increased PM_2.5_ concentration can cause serious harm to the human body, thus leading to negative externalities and even reduced life expectancy [2]. As China aims to improve its current air quality and to achieve peak carbon dioxide emissions by 2030 and carbon-neutral emissions by 2060, this environmental problem is extremely urgent in terms of pollution impacts. At the same time, China’s economic growth has slowed and is experiencing considerable downward pressure. As China is the largest developing country, its economic growth cannot be stalled by environmental issues [3]. The double pressure of environmental improvement and economic development has made it particularly important to implement a policy of sustainable development. In 2007, the *Opinions on Implementing Environmental Protection Policies and Regulations to Prevent Credit Risks* was put forward, marking the official start of a series of environment-oriented financing policies in China. According to the *2019 China Banking Social Responsibility Report* released by the Banking Association of China, by the end of 2019, the green credit balance of 21 major banks had exceeded 10 trillion CNY, making China the worldwide leader in green credit development.

Research on the performance of such an environment-oriented financing policy has been divided into two main categories: the impact on the economy and the environment.

In terms of economic growth, in theoretical qualitative research, the more developed a financial system is, the higher the total factor productivity will be; thus, the economy will eventually develop at a higher speed [4]. Slovakia [5] and Fangmin [6] believed that green finance can guide the flow of capital into green industry, thus promoting the development of green industry. Li Xiaoxi et al. [7] believed that green finance can provide financing channels for economic development and promote sustainable economic development. Wang Yao et al. [8] analyzed how green credit acts as an economic stimulus, for example, by leading to adjustments of the industrial structure and promoting the formation of green investment. Soundarrajan [9] concluded that green finance promotes economic growth through three channels: the formation and development of innovative technologies, the promotion of environmentally friendly enterprises and the formulation of effective trading strategies. Dong Wen [10] analyzed the mechanism and path of green credit on green economic growth in China and proposed that China should vigorously strengthen green credit policies and promote technological innovation and efficiency to promote green economic growth.

In terms of the findings of empirical and quantitative research, Ning Wei [11] found that the scale and resource allocation efficiency of green finance hinder economic development, so green finance has a negative effect on economic development. Qiu Haiyang [12] analyzed the influence of green finance on economic growth, which is manifested as a promoting effect, and proposed improving the green financial system and innovating green financial products. Pei Yu [13] took Zhejiang Province, China, as a sample to analyze the relationship between green credit input and regional economic growth, and the results showed that green credit promoted regional economic growth and that economic growth was a necessary condition for sustainable green credit input. Xu Sheng [14] found that green credit affects the industrial structure through the capital and channels of enterprises and has a significant impact on the adjustment and upgrading of the industrial structure. Xie Tingting [15] found that green credit plays a positive role in promoting the growth of the green economy. Liu Xia [3] believed that the investment amount of green credit plays an active role in promoting regional economic development, and the more advanced the industrial structure is, the stronger the promotional effect will be. Ding Jie [16] found that green credit did not improve but rather decreased total factor productivity.

In terms of theoretical qualitative research on environmental improvement, Mac [17] established the “pollution sanctuary hypothesis”, which posits that environmental regulation will make polluting enterprises gradually move from areas with stronger regulations to areas with weaker regulations, thus improving the environment of the former. Faulkender [18] analyzed the impact of green finance on environmental protection from the perspective of the capital leverage of enterprises and believed that green finance would enable environmentally friendly enterprises to obtain more capital through financing allocation and promote their expansion of production. Paola D Orazio [19] dynamically simulated traditional banks and green macro- and microenvironments through an agent model. The results showed that green finance plays a positive role in improving the environment. When green finance is compatible with consumer environmental quality consumption, environmental technology diffusion will be significantly increased, and green finance has a promoting effect on the environment. Mei Guoping [20] studied the dynamic relationship between green credit and energy-saving enterprises based on micro empirical data, and the results showed that green credit has a significant energy-saving effect. Wenyang et al. [21] combined economic growth theory with corporate social responsibility and established an endogenous growth model that included two types of enterprises, pollution and environmental protection, and the banking sector, proving that banking institutions should vigorously issue green credit and assume environmental responsibility.

In terms of empirical and quantitative research, Artur Tamazian [22] conducted a study on the relationship between finance and environmental quality, showing that financial development can effectively reduce pollutant emissions to improve environmental quality. Xu Helian [23] tested the impact of green finance on carbon dioxide emission intensity, and the results showed that both green investment and green insurance had a negative impact on carbon dioxide emissions. Tole et al. proposed the "pollution haven hypothesis" and found that environmental regulatory policies will gradually drive out heavily polluting enterprises [24, 25]. Han Liyan [26] established a four-factor model including environmental factors, and the results showed that the stocks of green environmental protection enterprises had excess returns; that is, there were green incentives in China’s stock market to encourage investment in the stocks of the enterprises so that corporate funds continued to flow into environmental protection projects. Fang Jianguo [27] established the first-level indicators of the four aspects of green credit. Green finance can save energy and reduce emissions, thus improving environmental quality. Shen Tao [28] studied the energy consumption effect of green finance pilot projects in Zhejiang and Guangzhou in China, and the results proved that green finance reduced the energy consumption per unit output value. Zhu Xiangdong [29] found that green finance has a spatial spillover effect that can be coordinated with and complement environmental regulations to achieve haze control horizontally by optimizing the industrial structure.

In summary, existing studies on the performance of green credit have the following main shortcomings.

First, although China is the largest issuer of green credit, the development of green credit is still unbalanced among regions. Most research has been aimed at parts of China, such as the northwest region [30], Beijing-Tianjin-Hebei [31, 32], the six central provinces [3], and Zhejiang Province [13], rather than the country as a whole. There has been little research on the overall implementation of performance assessment. China is a large country that covers an area of 960 square kilometers, leading to a certain difficulty in understanding the influence of green credit on China. Second, green credit improves the economy mainly through improving the industrial structure, encouraging green investment and stimulating green product consumption; there has been no research indicating whether other channels exist. The *Green Guidelines* (2008) issued by the People’s Bank of China point out that the implementation of green credit is intended mainly to limit the credit financing of enterprises that pursue the “two high and one surplus” approach (high pollution, high energy consumption and production capacity surplus) to reduce pollutant emissions and improve environmental quality. Therefore, this paper holds that green credit has an unintended positive impact on economic improvement through improving air quality, and evaluating the impact of environmental improvement on the economy through an empirical test of “Lucid Waters and Lush Mountains Are Invaluable Assets” is of great significance. This concept was first put forward by Chinese General Secretary Xi Jinping with the aim of establishing a sustainable economic development model and pursuing environmental improvement while achieving economic development, which implies the positive externality of environmental improvement on economic growth. Verification of the truth or falsity of this externality has been performed only theoretically [8, 9], and very few empirical studies have been based on data.

Addressing the lack of existing research is also an innovation of this paper. We use the chain-mediated model to evaluate the overall performance of green credit since it was issued in China, and it is not limited to local areas. Next, this paper estimates that since the issuance of green credit in China, in addition to industrial structure adjustment and green technology innovation, there is another way to affect economic growth, the positive externality of the improvement of the air environment. Such a path has been neglected by existing studies. The improvement of the air environment to achieve economic growth is a requirement of sustainable development and is of great importance. Similarly, in the analysis of subregional heterogeneity, due to the influence of regional differences and diminishing marginal effects, the promotional effect of green credit on different regions differs significantly. For example, the influence on the western region is greater than that on the eastern and central regions. In the treatment of the endogeneity problem, the first-order and second-order lag items of the core explanatory variables are taken as generalized method of moments (GMM)-type instrumental variables to re-estimate the model to alleviate the endogeneity problem. The coefficient signs of the core explanatory variables do not change and remain significant. The robustness test is divided into two parts: in the first part, the PM_2.5_ air quality measurement index is replaced by CO_2_ emissions, and in the second part, bootstrap sampling is carried out to correct the estimation deviation that may appear due to stepwise regression. The results show that the coefficients remain significant and in the same direction; thus, the effect is robust.

## Methods

### Theoretical analysis

#### Environmental quality and economic growth

In the traditional model of economic development, the relationship between the economy and the environment is contradictory. Models of economic development, such as that of Solow, did not include environmental factors. In 1995, Porter combined the environment with total factor productivity and found that external environmental regulations would force enterprises to improve their own technological innovation to improve total factor productivity [33] and promote output growth. For example, in the Cobb-Douglas production function, coefficient A on the right side of the equation increases. However, there are still different opinions in academic circles on whether the Porter hypothesis is valid [34] or invalid [16]. Grossman and Krueger [35] studied the relationship among four environmental indicators (urban air pollution, oxygen status, fecal pollution and heavy metal pollution in river basins) and economic growth and found that there was no clear evidence that environmental growth steadily declined with economic growth. Rather, in most cases, the relationship between environmental quality and economic growth is U-shaped; hence, they were the first to propose the environmental Kuznets (EKU) curve. Later, many studies gradually confirmed this point of view and verified the relationship between the environment and economic growth.

In addition, the “double dividend” hypothesis holds that an environmental tax can improve environmental quality and distort the tax system to achieve economic growth [36]. However, the validity of this hypothesis is also controversial. Zhou et al. [37] simulated microeconomic data for Chongqing and found that the collection of an environmental tax would have a negative impact on increasing family income and economic growth; thus, there was no “nonenvironmental dividend” effect. They concluded that the double dividend hypothesis of environmental taxation was invalid. Based on regional differences, Ciaschini [38] simulated the dual regional economic conditions in different regions of Italy and found that the first environmental income dividend appeared in the overall economic system, while the second economic dividend appeared in the northern and central regions, which was an effect of the complex socioeconomic structure. Thus, the above studies have shown that the environmental impact on different regions may vary greatly.

#### Environmental quality, human health and labor supply, and labor productivity

The impact of environmental problems on human health has always been a category of medical research. However, with the development of environmental economics, the impact of environmental quality on human health has gradually entered the field of economics. Chen et al. [2] studied the Huai River policy for indoor heating and found that every 184 μg/m^3^ or 55% increase in total suspended particulates (TSPs) in the northern region resulted in a 5.52-year decrease in life expectancy and an increase in cardiopulmonary mortality. Thus, this policy has resulted in a staggering loss of more than 2.5 billion life years in the Huai River, thus quantifying the negative externality of environmental pollution. Although this study is somewhat controversial, Ebenstein [39] came to the same conclusion after improving the monitoring data: heavy pollution significantly increases the probability of heart and lung disease, thereby reducing life expectancy. Richard [40] found that in the UK, the human health level was affected by environmental quality, and the human health level was related to labor participation, which had a significant impact on economic output. Therefore, he concluded that environmental quality has an impact on economic output.

Environmental quality has an important impact on human health, and the level of human health has an impact on the willingness and efficiency of residents to work. Regarding labor supply, Ostro [41] found a certain correlation between the concentration of particulate matter and labor loss. Hausman [42] found that an increase in air pollution was related to an increase in labor supply loss. Pönkä [43] found that an increase in the concentration of SO_2_ and NO_2_ in the air and a decrease in temperature would lead to an increase in the number of upper respiratory tract infections as well as an increase in the incidence of absenteeism in day care centers, schools and workplaces. Koundouri [44] found that shallow drinking water wells in Bangladesh were widely contaminated by arsenic, and such pollution resulted in a reduction of labor supply by more than 8%. In addition, several studies have shown that environmental pollution can lead to higher absenteeism among workers, thus affecting labor supply [45–48].

In addition to labor supply, environmental quality has an impact on labor productivity during working hours. Crocker et al. found that ozone levels far below federal air quality standards had a significant impact on the productivity of agricultural workers [49, 50]. Hanna et al. found that the reduction of environmental pollution can bring several potential benefits, including the improvement of health status and labor productivity [51, 52]. Frankenberg [53] evaluated the impact of Indonesia’s fire on the air environment and found that elderly people in disaster areas had more difficulties in daily life than those in non-disaster areas. Such difficulties were caused mainly by the negative impact of haze on human daily labor productivity.

Mechanism analysis shows that environmental quality can affect labor supply or labor productivity by influencing human health, and labor supply and labor productivity are the major factors affecting economic growth. The issuance of green credit is aimed mainly at restricting the financing of heavily polluting enterprises, forcing them to reduce pollutant emissions, improve their industrial structure and realize green innovation technology to improve the overall environmental quality level and achieve sustainable development. Therefore, it is theoretically possible for the issuance of green credit to increase labor supply or labor productivity through environmental improvement, thus promoting economic growth. Such a path is a process of unconscious internalization of positive externalities.

### Hypotheses proposed

Based on the above mechanism analysis, we find that the improvement of air quality can affect two aspects: labor supply and labor productivity. Labor supply and labor productivity are two important factors affecting regional economic growth. China still relies on labor supply to improve economic growth. In 2021, China is vigorously encouraging childbearing in order to obtain the "demographic dividend" brought by the increase of labor supply. In the context of the increasing amount of green credit development, it is particularly important to investigate whether the issuance of green credit in China can affect labor supply through environmental quality. In addition to labor supply and labor productivity, there is no literature showing other channels through which air quality affects regional economic growth. Therefore, environmental improvement may have a certain positive externality that can promote economic growth through labor supply or labor productivity. The issuance of green credit is intended mainly to improve environmental quality and realize sustainable development. Can the positive externalities of environmental improvement simultaneously promote China’s environmental improvement and achieve economic growth? Alternatively, “Lucid Waters and Lush Mountains Are Invaluable Assets” is a good practice. How does the externality of green credit for environmental quality improvement affect labor supply and labor productivity?

**Fig 1:**
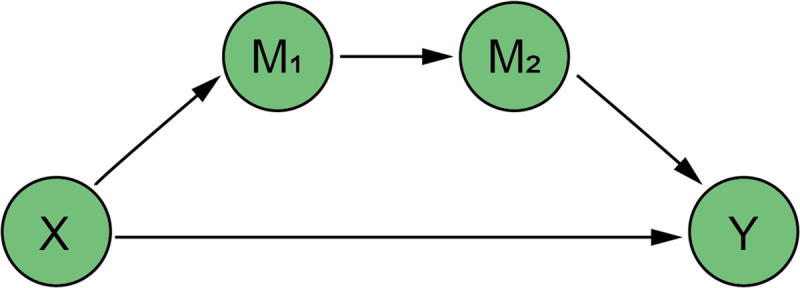
Chain-mediated path 1: Air quality, labor supply.

**Fig 2:**
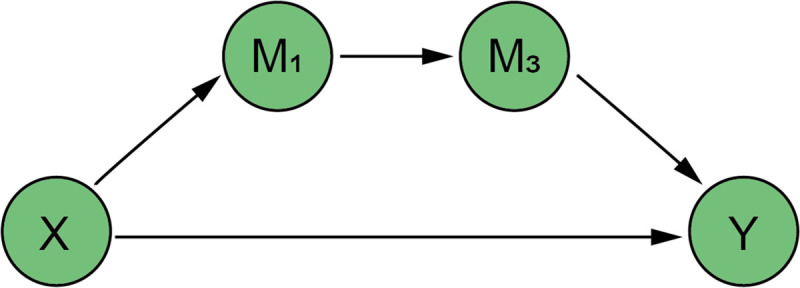
Chain-mediated path 2: Air quality, labor productivity.

In the existing literature, the influence of air environmental quality on labor supply and labor productivity has mostly been observed in the United Kingdom [40], the United States [41, 42, 50], Finland [43], Bangladesh [44], Mexico [52], Indonesia [53] and other regions, while a few are located in China [2, 39]. The impact is not the same in different areas. In China, it is not clear which channel it is, namely, whether the externality of green credit for environmental quality improvement affects labor supply or labor productivity. In particular, there are no comprehensive study based on it, which regard China as a whole part. For the above reasons, this paper proposes two hypotheses in combination with the chain-mediated model to explore the exact environmental and economic performance of green credit, so as to promote the further development of green credit in China (Figs 1 and 2):

*H1a*: *The issuance of green credit will not affect labor supply through air environmental quality and then affect the level of domestic output*.
*H1b*: *The issuance of green credit will affect labor supply through air environmental quality and then affect the level of domestic output*.
*H2a*: *The issuance of green credit will not affect labor productivity through air environmental quality and then affect the level of domestic output*.
*H2b*: *The issuance of green credit will affect labor productivity through air environmental quality and then affect the level of domestic output*.

## Model specification

### Data source

Data are selected from the China Industrial Statistical Yearbook, China Urban Statistical Yearbook and Atmospheric Composition Analysis Group. The linear interpolation method is used to fill in missing values for some years and provinces. Since the Opinions on Implementing Environmental Protection Policies and Regulations to Prevent Credit Risks was issued in July 2007, to eliminate the influence of the delayed implementation of the policy, the data selection starting time is 2008, the closing time is the latest edition of the China Industrial Statistical Yearbook 2017, and the recorded content is the data of 2016. Since there are many years of missing data in Tibet, interpolation and filling cannot be carried out. Therefore, Tibet is removed from the 31 provinces of China, and the final data are the panel data of 30 provinces in China from 2008 to 2016. The core variables are explanatory variables, explained variables and intermediary variables (M1, M_2_/M_3_).

### Explained variable

Economic growth: The per capita gross domestic product (GDP) of each province is selected as the index to measure the economic growth of each province. Although there are various deviations or inadequacies in measuring economic growth, GDP is still an important indicator of economic growth [54].

### Explanatory variables

Although China has the largest issuance of green credit, the development of green credit itself is not mature in China. One obvious feature is that there is no direct index to measure the issuance of green credit, and the disclosure of credit information is limited. Therefore, there are several methods to construct the index of green credit issuance in China: Lian Lili [55] measured green credit in the form of dummy variables, and the value of the variable is 0 before the issuance year and 1 after that. Xu Sheng [14] measured the issuance of green credit with the balance of China’s energy conservation and environmental protection projects and service loans with the balance of financial institutions’ loans at the end of the year. Xie Tingting [15] measured green credit by subtracting the interest expenditure of industries from the interest expenditure of six high-energy consuming industries in the total expenditure of interests. Guo Wenwei and Liu Yingdi [56] used the ratio of green credit balance to total loans and the ratio of "two high and surplus" loans to total loans as green credit to measure the degree of green credit business of major banks. Fang Jianguo [27] adopted the sum of the proportion of the balance of standardized energy conservation and environmental protection projects in the balance of loans of financial institutions and the proportion of the interest expenditure of high-energy consuming industries in the total industrial interest expenditure as the issuance of green credit. Wang Kangshi [57] measured green credit by using the flow of green credit resources from polluting industries to environmental protection industries.

The object of this paper is the national level, and the data of major banks or financial institutions are not sufficient. Considering the integrity of the data and the panel data of each province used in this paper, the ratio of interest expenditure of six high-energy industries to green credit issuance is selected for measurement. The method of measurement is “1- the proportion of interest expenditure of the six high-energy consuming industries (petroleum processing and coking and nuclear fuel processing, chemical raw materials and chemical products manufacturing, ferrous metal smelting and rolling processing, nonferrous metal smelting and rolling processing, nonmetallic mineral products industry, and production and supply of power and heat).”

GreenCredit=[1–(Interestdisbursementsforsixhigh‐energyindustries/Industrialinterestexpenditure)]*100% (1)

### Intermediary variables

Air quality (M_1_): To simplify the analysis, PM_2.5_ concentration is taken as the proxy variable of air quality. In some studies, each index (SO_2_, solid waste emissions, etc.) is artificially weighted, and the air environmental index that is compiled is highly subjective. The PM_2.5_ concentration is selected as the proxy variable of environmental quality to avoid such subjectivity, and the PM_2.5_ concentration itself is a comprehensive index that can be used to measure air quality. PM_2.5_ concentration is also the major source of industrial pollution emissions, and concentration changes in PM_2.5_, as suspended particulate matter, have a great impact on human health [2, 39, 58]. Therefore, the regional average PM_2.5_ concentration in the Atmospheric Composition Analysis Group is used as a regional air quality indicator in this paper.

Labor supply quantity (M_2_): The quantity of labor supply varies from industry to industry. However, since this paper measures the overall situation, the number of employees in urban units at the end of the period is adopted to measure the labor supply quantity in each province.

Labor productivity (M_3_): the ratio of income to employees is used to measure labor productivity according to Liu [59]. The annual GDP of each province/number of employees in urban units at the end of the year is adopted to measure the labor productivity of each province to compensate for differences in labor productivity among different industries.

### Control variables

The control variables are selected according to the characteristics of the province:

Urbanization: The urbanization rate of a province will affect the urbanization progress and thus the development degree of urban industrial enterprises [60], while PM_2.5_ is affected largely by the number of urban industrial enterprises.

Technological innovation (research and development (R&D)): The intensity of R&D input is selected as the control variable to control the impact of technological innovation on economic growth [61]. Investment intensity is equal to the proportion of provincial R&D expenditure in a province’s GDP.

Human capital: Human capital is measured by the average number of years of education received in each province [60]. Average years of schooling is equal to the proportion of uneducated population×0+ proportion of population with primary school education×6+ proportion of population with junior middle school education×9+ proportion of population with senior high school education×12+ proportion of population with college education or above×16.

Foreign direct investment (FDI): The economic impact of FDI is taken as a control variable [62].

### Descriptive statistics

The dataset is short panel data in which N(30)>T(9), and the influence of the time trend is not the major factor that causes differences in data within the group. Such a difference is more likely caused by differences in individual factors. According to the above descriptive statistics, the individual differences are caused mainly by regional differences. The most significant differences are in per capita GDP, labor supply, R&D input intensity and FDI (Table 1), of which the western region accounts for only 46.28%, 40.79%, 49.71% and 15.92%, respectively, of the amount accounted for by the eastern region, all less than 50%. Moreover, the regional inequality of several other indicators has eased. This phenomenon is caused not only by differences in industrial structures in the eastern and western regions but also by economic development, which explains the necessity of heterogeneity analysis.

**Table 1:** Descriptive statistics of each variable by region.

	Notes	Variable	Domestic	Eastern	Central	Western
Explained variable	Per capita GDP	Pergdp	3.883	5.236	3.539	2.423
Explanatory variable	Proportion of green issuance	Gd	45.129	52.663	47.793	32.386
Intermediary variable	Labor supply	Labor	5,088,349	6,967,284	4,829,177	2,842,275
	Labor productivity	Producti	34.735	37.669	36.524	29.033
	PM_2.5_ concentration	Geopm2.5	39.521	44.181	41.710	31.117
Control variable	Urbanization rate	Urban	0.541	0.638	0.505	0.449
	R&D investment intensity	R&D	1.490	2.088	1.144	1.038
	Foreign direct investment	FDI	720,167.3	1,222,870	575,371.3	194,693
	Average years of education	Edu	8.852	9.361	8.849	8.176
Sample size			270	108	81	81

The stepwise regression method adopted by Baron [63] is used to estimate the above chain-mediated model; that is, the existence of a chain-mediated effect is judged according to the significance of the coefficients. However, Wen Zhonglin [64] believed that testing only the mediating effect based on stepwise regression would lead to bias, and stepwise regression would increase the probability of errors in the first type of statistical inference. The mediating effect testing strategy selected in this paper first tests the validity of the above hypothesis according to the stepwise regression method and then further tests the existence of a chain-mediated effect by bootstrap sampling in the robustness test. According to the chain-mediated model of green credit issuance on economic growth, a benchmark model is set to test the impact of green credit issuance on economic growth:

$$Pergdp_{it}=\alpha_{0}+\alpha_{1}Gt_{it}+\eta_{2}X+\mu_{i}+\delta_{t}+\varepsilon_{it}$$ (2)

*Pergdp*_*it*_ is the explained variable and represents the per capita GDP of the *i*th province in year *t*; *Gd* is the core explanatory variable and represents the green credit issuance of the *i*th province in year *t*; X represents the control variables, including the urbanization rate of each province, investment in R&D, FDI, and average years of education; *μ_i_* is the fixed effect of province; *δ_t_*s is the fixed effect of time; and *ε_i_* is the residual item.

According to the above intermediary mechanism analysis and hypothesis setting, green credit issuance may improve labor supply or labor productivity through the improvement of environmental quality to promote economic growth.

To test the promotional effect of this mediation mechanism, this paper sets the following model:

$$Geopm_{2.5}=\varphi_{0}+\varphi_{1}Gt_{it}+\eta_{2}X+\mu_{i}^{'}+\delta_{t}^{'}+\varepsilon_{it}^{'}$$ (3)

$$\ln labor/\ln producti=\lambda_{0}+\lambda_{1}Geopm2.5+\eta_{2}X+\mu_{i}^{''}+\delta_{t}^{''}+\varepsilon_{it}^{''}$$ (4)

$$Pergdp_{it}=\beta_{0}+\beta_{1}Gt_{it}+\beta_{2}\ln labor/\ln producti+\eta_{3}X+\mu_{i}^{'''}+\delta_{t}^{'''}+\varepsilon_{it}^{'''}$$ (5)

where *Geopm*_2.5_ and ln*labor*/ln*producti* are intermediary variables representing the regional average PM_2.5_ concentration and labor supply or labor productivity of province *i* in year *t*. Eqs (2)–(5) list two intermediary variables of air environmental quality and labor supply or labor productivity and a regression equation of economic growth driven by one intermediary path. X is the control variable, representing a number of variables of independent and green credit issuance on economic growth. $\mu_{i}^{'}、\mu_{i}^{''}、\mu_{i}^{'''}$ is the fixed effect of province, and $\delta_{t}^{'}、\delta_{t}^{''}、\delta_{t}^{'''}\frac{}{}$is the fixed effect of time. According to the chain-mediated model, the mediating effect size is *φ*_1_*λ*_1_*β*_2_, and the direct effect is *α*_1_. According to Eqs (2)–(5), the chained mediating effect is determined by successively testing the regression coefficients. If the coefficient in Eq (3) is significant, it indicates that there is an overall effect that is the sum of the direct effect and the chain-mediated effect. If the coefficient in Eq (5) is significant, it indicates that there is a direct effect.

## Empirical results

According to the above analysis and data, the random effect model and fixed effect model were first selected for the Hausman test. The results show that the Hausman statistic is 64.95; thus, the null hypothesis that a random effect should be selected is strongly rejected. Therefore, the fixed effect is adopted to perform regression on Eqs (2)–(5) in turn.

### The national level

Models (1)-(4) are Chain-Mediated Model 1 (air environmental quality, labor supply), and Models (1), (2), (5), and (6) are Chain-Mediated Model 2 (air environmental quality, labor productivity). The coefficient of *Gt* in regression (1) reflects the direct effect of green credit on per capita GDP; that is, if the proportion of green credit issuance increases by 1%, the average per capita GDP will increase by approximately 160 yuan per year at the national level (Table 2).

**Table 2:** Estimation of the mediating effect at the national level.

	(1)	(2)	(3)	(4)	(5)	(6)
Method	FE	FE	FE	FE	FE	FE
Variable	Pergdp	Geopm_2.5_	lnLabor	Pergdp	lnProducti	Pergdp
Gt	0.016**	-0.149***		0.016*		0.016*
	(0.008)	(0.058)		(0.008)		(0.008)
lnLabor				0.437*		
				(0.260)		
lnProducti						0.563**
						(0.321)
Geopm_2.5_			-0.007***		-0.004	
			(0.003)		(0.003)	
Urban	-0.391	-67.200***	1.673***	-1.283	0.066	-0.320
	(3.020)	(21.202)	(0.632)	(3.021)	(0.997)	(2.883)
lnR&D	0.226	-6.169	0.194**	0.120	-0.445***	0.489
	(0.398)	(2.607)	(0.092)	(0.409)	(0.092)	(0.439)
lnFDI	-0.005	0.618	-0.037*	-0.020	0.033	-0.024
	(0.135)	(0.689)	(0.023)	(0.133)	(0.029)	(0.133)
Edu	-0.568	-9.068	0.012	-0.608	0.619	-0.896
	(1.660)	(13.081)	(0.455)	(1.645)	(0.532)	(1.651)
Constant	4.627	95.770***	14.906***	-1.600	1.642	3.524
	(4.021)	(29.634)	(1.010)	(5.556)	(1.328)	(3.955)
N	270	270	270	270	270	270
R-squared	0.951	0.956	0.980	0.951	0.816	0.952
Number of id	30	30	30	30	30	30

*** p<0.01

** p<0.05

* p<0.1; robust standard errors are shown in brackets, as follows.

In terms of labor supply, according to Models (2), (3) and (4), the chained mediating effect is approximately 0.00046 (-0.149×-0.007×0.437); that is, every 1% increase in the proportion of green credit issuance can increase per capita GDP by approximately 4.6 yuan through the improvement of air environmental quality and the improvement of labor supply. The national per capita GDP from 2008 to 2016 was approximately 38,830 yuan, which translates into a percentage of approximately 0.012%. Thus, Hypothesis H1a is supported. The improvement of the air environment affects the labor supply in many ways. The labor provided by individuals is not only determined by the labor supply willingness of the population but also determined by the individual health level. For individual health, air environmental quality not only has a huge impact on human health but also increases the mortality rate caused by cardiovascular diseases and other diseases. In the long term, it will also reduce the average life expectancy of local people [2, 39]. Among them, the influence of TSPs in China is taken as an example. Every 184μg/ m^3^ or 55% increase in TSP in the northern Huai River area of China can reduce the life expectancy by 5.52 years and increase the cardiopulmonary mortality rate in this area [2], and PM2.5 belongs to the category of TSP. For individuals’ willingness to supply labor, the deterioration of environmental quality will also lead to a decrease in individuals’ willingness to supply labor, resulting in a higher rate of absenteeism [43, 44]. This is especially true in western China, where machines are less automated and mostly employ human labor. Compared with the central and eastern regions, the working pressure is lower, the attendance system is more flexible, and the individual subjective consciousness is stronger. Therefore, when air quality deteriorates, it is more likely to cause a decrease in the overall level of individuals’ disposition to supply labor, that is, a higher absenteeism rate.

In terms of labor productivity, the coefficient from air environmental quality to labor productivity, the core explanatory variable of Model (5), is not significant, so the improvement of air environmental quality has no obvious influence on the improvement of labor productivity. Thus, Hypothesis H2b is supported. The reason why improvements in environmental quality have little effect on labor productivity may be that traditional labor industries, such as those that require much physical exertion, are gradually being replaced by automation. Most positions requiring manpower are those with low labor consumption, and environmental improvement has no significant impact on positions with a higher automation degree. At the same time, the pollution sanctuary hypothesis [17] points out that polluting enterprises will move from places with strong environmental regulations to places with weak environmental regulations. The scale of green credit is gradually expanding in China. When environmental regulations such as green credit are implemented nationwide, most highly polluting enterprises are limited by heavy fixed assets and equipment and cannot transfer, which may lead to the phenomenon of delayed response, thus resulting in a decrease in productivity [16].

The above analysis at the national level shows that green credit issuance can affect labor supply through improvements in environmental quality. Labor supply is still an important factor affecting economic growth [65]. According to the results, this mediating effect accounts for approximately 2.875% (4.6/160) of the total effect of green credit in promoting economic growth. Existing studies show that green credit mainly promotes economic growth through the transformation and upgrading of industrial structure and the improvement of green technology innovation. However, the approach studied in this paper is the impact of environmental quality improvement on labor supply, which is not the main approach. Therefore, the mediating effect is far less than the effect brought by industrial structure adjustment and technological innovation on economic growth.

### Eastern, central and western regions

In the heterogeneity analysis, the effect of green credit issuance on labor productivity is still not obvious, so this part lists only the results for which the mediating variable is labor supply. Subregional regressions are performed on Eqs (2)–(5) in sequence for the eastern (Table 3), central (Table 4) and western (Table 5) regions of China. The results show that green credit has a stronger effect on promoting economic growth in western China. According to the chain-mediated effect model, the per capita GDP growth of green credit through the improvement of the environment and labor supply in western China is approximately 30.17 yuan (-0.324×-0.016×0.582), which is approximately 6.6 times greater than the national level. At the same time, the chain-mediated effect accounts for approximately 10.057% of the total effect of green credit on promoting economic growth, indicating that the effect of green credit on promoting economic growth in other ways, such as industrial structural adjustment and green technology innovation, is stronger in western China than at the national level. The reason may be related to the marginal diminishing effect. In the whole country, especially in the central and eastern regions, the industrial structure, industrial technology level and financial service system are all ahead of those in the western regions of China. Due to the law of diminishing margins, the effect of green credit on a relatively underdeveloped area is stronger than that on a relatively well-developed area. Some studies have also pointed out that on the whole, the impact of environmental regulation on China’s economic growth is stronger in the central and western regions than in the eastern regions [60]. In addition, the western region is not a concentrated area of the labor force, and the labor outflow phenomenon is serious. When external environmental regulation intensifies, some polluting enterprises will flee. The escape of those enterprises to some extent causes the return of the local labor force and improves the labor supply, thus increasing regional GDP. Obviously, such GDP growth does not conform to the concept of sustainable development [17]. According to the regression results in the western region, green credit can better reduce the PM_2.5_ concentration, so the issuance of green credit can better force the transformation and upgrading of such more “liquid” enterprises, rendering them unable to continue to escape.

**Table 3:** Estimation of the mediating effect of labor supply at the regional level in eastern China.

	(1)	(2)	(3)	(4)
Method	FE	FE	FE	FE
Variable	Pergdp	Geopm_2.5_	lnLabor	Pergdp
Gt	-0.003	-0.044		0.005
	(0.012)	(0.075)		(0.012)
lnLabor				0.768*
				(0.486)
Geopm_2.5_			-0.003	
			(0.004)	
Control Variable	√	√	√	√
Constant	-6.646	47.812	15.301***	-18.494*
	(8.721)	(55.447)	(1.948)	(11.443)
N	108	108	108	108
R-squared	0.960	0.968	0.985	0.961
Number of id	12	12	12	12

**Table 4:** Estimation of the mediating effect of labor supply at the central regional level.

	(1)	(2)	(3)	(4)
Method	FE	FE	FE	FE
Variable	Pergdp	Geopm_2.5_	lnLabor	Pergdp
Gt	0.001	-0.210		0.002
	(0.012)	(0.151)		(0.013)
lnLabor				0.144
				(0.510)
Geopm_2.5_			-0.004	
			(0.003)	
Control Variable	√	√	√	√
Constant	17.928***	97.257	13.343***	16.251**
	(6.269)	(79.442)	(1.674)	(8.655)
N	81	81	81	81
R-squared	0.954	0.960	0.962	0.954
Number of id	9	9	9	9

**Table 5:** Estimation of the mediating effect of labor supply in western China.

	(1)	(2)	(3)	(4)
Method	FE	FE	FE	FE
Variable	Pergdp	Geopm_2.5_	lnLabor	Pergdp
Gt	0.036**	-0.324***		0.030*
	(0.016)	(0.122)		(0.016)
lnLabor				0.582**
				(0.278)
Geopm_2.5_			-0.016**	
			(0.007)	
Control Variable	√	√	√	√
Constant	-11.708**	50.760	14.079***	-9.640***
	(5.732)	(44.022)	(2.537)	(6.742)
N	81	81	81	81
R-squared	0.935	0.941	0.976	0.939
Number of id	9	9	9	9

Based on the analysis of national-level and regional heterogeneity, green credit at the national level promotes the growth of regional per capita GDP through a chain-mediated path such as air environmental quality and labor supply, which has a small mediating effect, accounting for approximately 2.85% of the total effect. The effect in the central and eastern regions is not significant, while the effect in the western region is significant at approximately 6.6 times more than the national level. The research shows that green credit promotes the economy mainly through the transformation and upgrading of the industrial structure and the improvement of green technology innovation. However, the approach studied in this paper is the impact of environmental quality improvement on labor supply, which is not the main influence. Therefore, the mediating effect is far less than the effect of industrial structural change and technological innovation on economic growth.

### Endogeneity and robustness test

This section consists of two parts. The first part is the endogeneity problem of bidirectional causality that may exist in the model, and the second part is divided into two steps for the robustness test. The first step selects CO_2_ emissions to replace the air environmental quality index to estimate Eqs (2)–(5) in turn. In the second step, the bootstrap sampling estimation method is used to test the chain-mediated effect. The number of sampling times was 5000, and the confidence of the results reported was the upper and lower limits of 95%. Both parts show that the estimation results of the above model are robust.

### Endogeneity problem

The following relationship is assumed to be completely exogenous: the effects of green credit issuance on per capita GDP, namely, Eq (2); the effects of green credit issuance on PM_2.5_ concentrations, namely, Eq (3); the effects of PM_2.5_ concentrations on labor supply, namely, Eq (4); and the effects of labor supply on per capita GDP, namely, Eq (5). The above analysis indicates that the issuance of green credit can improve the per capita GDP of the region by affecting the air environment and labor supply. However, economic theory shows that there may be a mutually causal endogenous relationship between Eqs (2) and (5); that is, the explanatory variables and the explained variables in the same period will influence each other. Such an endogenous relationship will lead to the inaccuracy of coefficient estimation results.

To avoid such an endogeneity problem of bidirectional causality, the lagged first-order and second-order terms of the core explanatory variables of Eqs (2)–(5) are selected as GMM-type instrumental variables, and the control variables are assumed to be exogenous. The model is re-estimated to mitigate the inaccuracy of the endogenous relationship in the estimation of the conclusion. An additional second-order lag is selected as the instrumental variable because only a first-order lag cannot detect the validity of the instrumental variable when the number of instrumental variables introduced is greater than the original number of endogenous variables. Sargan statistics are used to detect whether the instrumental variable is valid. The second-order lag is still highly correlated with the current item, and the current residual correlation further weakens. The results of GMM estimation show that only the significance of the coefficient of the key explanatory variable is enhanced, and the sign of the coefficient does not change (Table 6). Sargan statistics show that all other instrumental variables of the model are valid except those in Models (1) and (4) at the significance level of 10%, rejecting the original hypothesis that “all instrumental variables are valid instrumental variables”.

**Table 6:** Estimation of the mediating effect of labor supply at the national level.

	(1)	(2)	(3)	(4)
Method	GMM	GMM	GMM	GMM
Variable	Pergdp	Geopm_2.5_	lnLabor	Pergdp
Gt	0.056***	0.415***		0.051***
	(0.014)	(0.118)		(0.013)
lnLabor				0.864***
				(0.220)
Geopm_2.5_			0.018***	
			(0.008)	
Control Variable	√	√	√	√
Sargan	0.010	0.902	0.391	0.010
N	210	210	210	210
Number of id	30	30	30	30

### Variable substitution

Here, we choose CO_2_ as the object of variable substitution, mainly for the following two reasons. First, CO_2_ emissions in the air will be transformed into CO when emissions are too high, and CO is strictly an air pollutant index, so CO_2_ will also pollute the air environment to a certain extent. Second, SO_2_ [61], NOx and common solid waste discharge [66, 67] have been used as the experimental objects of existing studies. If these pollutants are selected again here, repeated experiments will be performed, and similar results will be obtained. However, most studies do not take CO_2_ as the research object, and in the context of China’s "Carbon Peak" by 2030 and "Carbon Neutral" by 2060, taking CO_2_ emissions as the variable is more in line with the current background.

In this section, the PM_2.5_ concentration is replaced with CO_2_ emissions, and the above equations are then regressed. The provincial CO_2_ emission data are derived from the time series data of 30 provinces in China constructed by Shan et al [68]. The results show that the coefficients of the core explanatory variables of the replaced Models (2) and (3) are still significant, and the sign direction has not changed. At the same time, the size of the chain-mediated effect at the national level is estimated to be approximately 3.94 (-0.901×-0.001×0.437), which is close to the above estimated results when the PM_2.5_ concentration is used as the explanatory variable Table 7).

**Table 7:** Estimation of the mediating effect of labor supply at the national level.

	(1)	(2)	(3)	(4)
Method	FE	FE	FE	FE
Variable	Pergdp	CO_2_	lnLabor	Pergdp
Gt	0.016**	0.901***		0.016*
	(0.008)	(0.363)		(0.008)
lnLabor				0.437*
				(0.260)
CO_2_			0.001**	
			(0.0003)	
Control Variable	√	√	√	√
Constant	4.627	222.669***	14.372***	1.600
	(4.021)	(203.646)	(1.077)	(5.556)
N	270	270	270	270
R-squared	0.951	0.982	0.980	0.951
Number of id	30	30	30	30

### Bootstrap sampling

The SPSS built-in program Process, which is written by *Andrew F*. *Hayes* (*www.Afhayes.com*), is used for bootstrap sampling. Model 6 is selected to conduct bootstrap sampling simulation for the above chain-mediated model 5000 times, and a 95% confidence interval is reported. If the bootstrap sampling confidence interval contains 0, the effect is not significant. Note that Model 6 assumes that there are two mediation paths in addition to the model mentioned in this article: X→M_1_→Y and X→M_2_→Y. The first path is not applicable because the PM_2.5_ concentration may not have a direct impact on economic growth. Existing studies on the second path show that the increase in labor supply is caused largely by the adjustment of the industrial structure, so it is not the focus of this paper. This paper focuses on the chain-mediated path X→M_1_→M_2_→Y, where M_1_ and M_2_ are air quality and labor supply or labor productivity, respectively. No research has shown the existence of this path. To prevent the above incorrect estimation of labor productivity, a bootstrap sampling test is conducted for Hypothesis 1and 2, as shown in Tables 8 and 9. The results show that when bootstrap sampling is used for 5000 simulations, the chain-mediated model with labor supply does not contain 0 in the 95% confidence interval, and this effect accounts for approximately 12.96% of the total indirect effect; thus, H1b is supported. However, the chain-mediated model with labor productivity is invalid, and the improvement of air quality may indeed have no effect on labor productivity. Thus, H2a is supported.

**Table 8:** Bootstrap sampling for the mediating effect of labor supply.

	Effect of value	Boot Standard error	Boot CI The lower limit	Boot CI The upper limit	Percentage
Total indirect effect	0.0324	0.0058	0.0205	0.0432	100.00%
Green credit issuance →PM2.5 concentration → per capita GDP	0.0019	0.0043	0.0072	0.0096	5.86%
Green credit issuance → labor supply → per capita GDP	0.0264	0.0058	0.0151	0.0376	81.48%
Green credit issuance →PM2.5 concentration → labor supply → per capita GDP	0.0042	0.0016	0.0016	0.0079	12.96%

Note: Boot standard error, Boot CI lower limit and Boot CI upper limit refer to the standard error, 95% confidence interval lower limit and upper limit estimated by the bootstrap method sampling 5000 times with deviation correction, respectively, as follows.

**Table 9:** Bootstrap sampling test for the mediating effect of labor productivity.

	Effect of value	Boot Standard error	Boot CI The lower limit	Boot CI The upper limit	Percentage
Total indirect effect	0.0229	0.0051	0.0131	0.0328	100.00%
Green credit issuance →PM2.5 concentration → per capita GDP	0.0032	0.0028	0.0026	0.0086	13.97%
Green credit issuance → labor productivity → per capita GDP	0.0168	0.0040	0.0095	0.0250	73.36%
Green credit issuance →PM2.5 concentration → labor productivity → per capita GDP	0.0028	0.0017	0.0004	0.0062	12.23%

## Discussion and conclusion

### Discussion

Existing studies on green credit performance evaluation have focused on total factor productivity [16, 34], industrial restructuring [13, 14], and financial markets [69–71]. According to the *“Green Credit Guidelines”* issued by the China Banking Regulatory Commission, the implementation of green credit is intended mainly to improve the environment and realize sustainable development. Therefore, the model is set to introduce PM_2.5_ concentration to quantify the positive impact on economic growth brought by the improvement of air quality.

Based on existing studies, this paper estimates that since the issuance of green credit in China, in addition to industrial structural adjustment and green technology innovation, another new way to affect economic growth, namely, the positive externality of air quality improvement, can effectively promote the improvement of labor supply to achieve regional per capita GDP growth. Because a positive environmental externality is not the main approach, the chain-mediated effect is relatively small for the total effect, but it does exist. The results connect the improvement of the environment and economy, representing both an empirical test of the validation of the green credit policy and a practical proof of “Lucid Waters and Lush Mountains Are Invaluable Assets”. They also verify the EKU—the relationship between environmental quality improvement and economic growth is positive, not contradictory.

## Supporting information

S1 Table For data collection. (XLSX)

S2 Table For the calculation of green credit index. (XLSX)
